# Croupous Pneumonia

**Published:** 1892-01

**Authors:** R. M. Cunningham

**Affiliations:** Birmingham, Ala.


### Southern Medical Record.

A MONHLY JOURNAL OF MEDICINE AND SURGERY.

VOL. XXII. ATLANTA, GA., JANUARY, 1892. No. 1.

### Original Articles.

### CROUPOUS PNEUMONIA.

BY R. M. CUNNINGHAM, M. D., BIRMINGHAM, ALA.

There is, perhaps, no disease of more practical importance to the physician than croupous pneumonia.

According to Juergensen, about "66 per cent. of the total mortality is due to this disease, and about 12.7 per cent. of the mortality from internal diseases."

"Pneumonia occurs in all degrees of latitude, and does not appear to be more frequent in one latitude or climate than in another." (Ziemsen's Cyclopedia, vol. v., p. 12.

These two facts alone make the disease one of vast importance to every physician.

Notwithstanding these facts, there seems, even at this time, some confusion in the nomenclature of the various forms of pneumonia, their essential anatomical, pathological and etiological differences. The two forms of pneumonia most generally encountered in practice are the croupous and catarrhal. The same tissue is involved in both, to-wit: The intercellular air passages of Rainey, or the air sacs of Waters, and the cells or alveoli that open into them—collectively called the vesicular structure of the pulmonary parenchyma. That we may be more clearly understood, I beg your pardon for refreshing your minds for the moment on anatomy.

The bronchial tubes, after being reduced by dichotomous division—as a rule—to the diameter of 1-50 to 1-30 of an inch, generally from 4 to 9 to the lobule, change their structure and continue as "irregular passages through the substance of the lobule, their sides and extremities being closely covered by numerous sacular dilatations, the air cells." They are from "1-200 to 1-70 inch in diameter, *being largest* on the *surface* at the free borders, and at the apex; and smallest in the interior." Their walls are formed by the interlacing of the longitudinal elastic bundles and fibrous tissue; the muscular fibres disappear, and the mucous membrane becomes thin and delicate and lined with a layer of squamous epithelium. The latter membrane lines the air cells, and forms, by its reduplications, the septa intervening between them."

The ramifications of the pulmonary artery and the beginning of the pulmonary veins, all forming fine and delicate plexus between these structures, form with the air sacs and cells, the essential parenchymatous structure of the lobules, and these, in turn, form the lobes, and the lobes the lungs.

The essential anatomical lesions of both these forms of pneumonia are found in these structures, but differing widely in their distribution, extent, causation and pathology.

I submit, in this connection, the opinions of two of America's most distinguished authors. Loomis says: "Croupous pneumonia is an acute general disease with a characteristic local pulmonary lesion. Anatomically considered, it is an acute inflammation of the vesicular structure of the lungs, resulting in infiltration of the alveoli with inflammatory products, which renders them impervious to air. This condition is known as hepatization."

Pepper says: "Catarrhal pneumonia is an inflammation of the parenchyma of the lungs, frequently bilateral and affecting scattered groups of lobules, which may, however, coalesce, so that considerable areas of lung-tissue become continuously involved. This anatomical distribution explains the name lobular as opposed to that of the lobar or croupous form."

Thus, according to these authors, the essential lesions of both forms of pneumonia consist in inflammation of the "vesicular structure," or "parenchyma" of the lungs. But this inflammation, in its causation, distribution and extent, nature, course, products and results, differs so widely in the two forms that in reality they have almost nothing in common except the character of tissue involved.

I submit, in parallel columns, some of the most important features of the two forms:

CROUPOUS PNEUMONIA.

1. An acute general constitutional disease, caused by a specific materies morbi, possibly the pneumococcus of Freedlander or Franklin coccus; at any rate, a germ of some description, probably not as yet fully demonstrated; generally primary, and when occurring as an intercurrent affection, is coincidental or possibly determined by the physical condition of the patient, acting either as a predisposing or exciting, but not as the *essential cause*, and having a characteristic local pulmonary lesion, to-wit: "inflammation of the vesicular structure" as its "anatomical sign."

2. Self-limited, running, as a rule, a typical course, usually terminating by crisis, in cases that recover, with complete restoration of the inflamed structures to their normal condition.

3. The inflammation, as a rule, simultaneously involves the lobules of a whole lobe, or a whole lung, or consecutively the lobes of one lung, sometimes one or more lobes of both lungs. It is therefore "lobar," and is usually unilateral.

4. The inflammation is characterized by well marked stages: (a) congestion of the pulmonary and bronchial circulation; and (b) red hepatization or the exudation in o the alve oli of a highly fibrinous material, holding in its meshes red and white blood corpuscles, a few altered epithelial cells, filling the alveoli and partially distending them, so that the whole lobe, or lung, is usually enlarged, the contents seldom extending into the terminal *bronchioles* or *lobular tubes*, and rendering the affected area airless; and finally (c), the retrograde physiological metamorphosis of this exudation, and its ultimate absorption, if life is prolonged, or rarely in local abscesses, purulent infiltration, or fibroid development, or gangrene.

5. The secondary pleurisy as a rule,

CATARRHAL PNEUMONIA.

1. An acute, subacute or chronic local disease, not generally constitutional nor infectious, consisting of a catarrhal inflammation of the vesicular structure, or pulmonary parenchyma, always secondary, being due, as a rule, to bronchial catarrh, is often a complication, therefore having for its essential cause some disease having for one of its anatomical signs catarrh of the bronchi, or the result of primary bronchitis, or may result, simultaneously, with bronchial catarrh, having a common cause.

2. Is not self-limited, runs an atypical course, seldom or never ends by crisis, and, as a rule, more or less permanently, in recovery, leaves behind it permanent lesions.

3. Inflammation, as a rule, involves disseminated lobules of a lobe, seldom the whole of it, and practically never simultaneously all the lobules of a lobe, a lung, or both lungs, is therefore lobular and is usually bilateral.

4. While there are stages of congestion and exudation, they are not so typical. There is usually associated in the lung tissue "congestion, œdema, emphysema, collapse and pneumonic consolidation." The morbid product filling the alveoli is more nearly allied to the secretion of a catarrh than the exudate of a phlegmonous inflammation. It consists mainly of the catarrhal contents of the bronchioles drawn into the alveoli by suction; of epithelial cells from the walls of the alveoli, of leucocytes and rarely red blood corpuscles and fibrillated exudation. The main difference in the inflammatory products is less fibrinous material and blood corpuscles, especially the red, and more of the epithelial elements.

5. The secondary pleurisy circumscribed, as a rule resulting in permanent circumscribed adhesion. The bronchitis may be, and usually is, when present, is of corresponding area with the pulmonic inflammation. The bronchitis, when present, confined to the pneumonic area, and is always secondary to the pneumonia, and rarely involves deeper structures than the mucous membrane, and does not lead to peri-bronchial inflammation nor bronchiectasis

6. Prevails, as a rule, as endemics or epidemics, at all times, seasons, latitudes and countries, occurring often without apparent cause, and leaving in the same way. These endemics and epidemics are characterized by mildness or severity the same as other infectious diseases. It seldom occurs sporadically, and is never *produced by exposure to cold.*

7. As a rule, there is no correspondence between the extent of the local lesion and the constitutional symptoms; the mode of death, as a rule, is asthenia or heart failure.

general, is primary to the pneumonia, and causes the latter. It may involve the entire structure of the tube and peri-bronchial structures leading to fibroid development of the connective tissue, and therefore to bronchiectasis.

6. Never occurs as an endemic or epidemic, except when due to endemic or epidemic diseases having bronchial catarrh as an anatomical lesion or sign, or frequently complicated by bronchial catarrh, and geographically corresponding with the latter disease when occurring as a primary disease. It often occurs sporadically and is the result generally in such cases, of cold, the latter first producing bronchial catarrh.

7. As a rule, the constitutional and local symptoms are measured by the extent of the local lesion. The mode of death is by apnœa.

There are many other differences, but the above, I think, is sufficient to demonstrate the radical difference between the two forms of pneumonia. No death certificate is complete by assigning the cause of death to "pneumonia," without a qualifying adjective giving the form.

My object in reading this paper is to give my experience in the treatment of some two hundred cases of croupous pneumonia occurring among the convicts at the prisons at Pratt mines. The experience covers a period of about eight and a half years. It is not my purpose in this paper to burden you with statistics or details, but will discuss the subject in a general way, leaving the statistics for a future paper.

These convicts are worked in the mines, and at work incident to mining. Therefore, we have "inside" and "outside" men.

That you may be informed as to the sanitary condition of the prisons, and the personal hygiene of the convicts, I will briefly give you an outline of these conditions, as they were eight years ago, and as they are now. Eight years ago the sanitary condition of the prisons and the hygiene of the convicts were just about as bad as could be. Deficient in cubic space, light and ventilation, filth, dampness and overwork were the main features of the bad hygiene. At present, and for the last six years, these conditions have been greatly improved, so that at the present time, in the language of Dr. Wines, Secretary of the National Prison Association, we have "the best cheap prisons in the United States." The sanitary condition during the past three years has been most excellent. The morbidity and mortality have been greatly reduced. For the five years ending October 1st, 1883, the death rate was 12.66 per cent. per annum; from October, 1883, to the present time, about 5 per cent. per annum. This demonstrates the wonderful reduction in death rate during the last eight years.

THE RELATION OF PRISON LIFE TO CROUPOUS PNEUMONIA.

For twenty years, ending October 1st, 1889, croupous pneumonia was the cause of death in 19.33 per cent. of the deaths in the Alabama penitentiary, due to disease. It thus appears that croupous pneumonia has caused the death in nearly 20 per cent. of the whole number of deaths—three times as much as under ordinary circumstances, according to Juergensen. There can be no question, therefore, that prison life, so far as Alabama is concerned, predisposes to the development of croupous pneumonia. In fact, all prisons, both military and civil, as well as active military life, predisposes to this disease.

Thus, during the war between the States, out of every 1,000 deaths among United States white troops, pneumonia was the cause of death in 11.84 per cent., and of the United States colored troops of 20.13 per cent.—the latter just about the same as in the Alabama penitentiary. Also, in certain commands of the Confederate armies, 20.6 per cent. (See Medical and Surgical History of the Rebellion, Part III.)

The explanation of this fact is usually made by assigning it to unsanitary conditions, bad hygiene, and to bad physical condition. I so explained it in my report in 1883 and 1884, as physician of the penitentiary. Such an explanation, however, falls to the ground in view of the facts as I have found them at Pratt mines. Thus, from June, 1883, to October, 1884, of the whole number of deaths at the Pratt mine prisons, croupous pneumonia was the cause in 16.60 per cent; from October, 1884, to October, 1886, 11.47 per cent.; from October, 1886, to October, 1888, 27.27 per cent., and from October, 1888, to October, 1889, 40.54 per cent., the average for the whole period being 22.94 per cent.

We thus find that the percentage of deaths due to pneumonia has more than doubled under the greatly improved sanitary conditions. In fact, the more we have done to improve, the greater has been the fatality from this disease. All other diseases—diarrhœa, dysentery, typhoid fever, continued fevers, etc.—have been practically abolished; but croupous pneumonia, until the last year, has remained with us.

This is certainly anomalous, and in strong contrast with the usual explanation of the causes of this disease. I certainly can make no explanation.

There are certain facts, however, which throw some light on the question. Our statistics demonstrate that there is much more sickness among those recently received than there is among those who have been committed for some time. The mortality is also greater. Prior to 1884 the mining prisons were largely recruited from the plantation prisons and from the penitentiary at Wetumpka, thus receiving convicts who had become more or less habituated to prison life. Since that time most of the convicts come directly from the jails. Again, from June, 1883, to October, 1884, most of the cases were of a mild type, and did not occur in groups as an endemic. Since October, 1884, most of the cases have been unusually severe, and occurred as endemics. I, however, can offer no explanation for these facts. My experience has led me to regard the essential cause of croupous pneumonia as not being dependent on bad sanitary conditions for either its production or maintenance. While these conditions may aggravate the influence of the germ, the latter can be developed and live under opposite conditions.

THE RELATION OF MINING COAL TO CROUPOUS PNEUMONIA.

From October, 1868, to October, 1880, the convicts were worked mostly on railroads and farms. Since that time the majority have been worked in mines and work incident to mining.

During the first, or farming and railroad period, croupous pneumonia was the cause of death in 19.20 per cent. of the whole number of deaths due to disease; and during the second period, *i. e.*, mining, 19.46 per cent. We thus see that there has been practically no increase since the convicts were put in the mines.

Again, an analysis of the cases at Pratt mines show a larger percentage among the outside convicts than among those who work in the mines. Finally, during the past eight years at Pratt mines, I have not observed, nor do I know of a dozen cases of croupous pneumonia occurring among the free miners, or free people (a population of about 4,000).

These facts are antagonistic to the generally accepted views of the profession, and to me is the most perplexing problem with which I have been confronted.

OUTLINE OF PERSONAL HISTORY OF THE CONVICTS

[Data obtained from examination of convicts when received from jail.]

About 80 per cent. were negroes ; 60 per cent. farm laborers; a large majority absolutely illiterate ; only about one-fourth had fine muscular development ; a little more than one-half had a good constitution. A bad and doubtful prognosis was made in 18 per cent. ; a little more than one third had bad family history ; a little more than one-third were actually diseased when received ; one-fourth were under twenty, and nine-tenths under forty years of age.

The above data were obtained from the examination of convicts when received from the jails, and are compiled from the in ividual examination of 1,229 convicts.

These facts demonstrate that the averag physical condition of the convict population of Alabama is not nearly so good as the outside population.

Of the 200 cases of croupous pneumonia, nearly all were negro males between the ages of twenty and forty years. Very few of the white convicts had the disease. Only onè female out of an average female population of about ten.

The records, here, at other prisons in Alabama, and of the late war, demonstrate that the negro, under more or less unfavorable environments, is particularly predisposed to pulmonary disease in all its forms, particularly pneumonic inflammation.

They also demonstrate that these diseases are much more fatal among negroes than among the whites. The only explanation that I can offer is, that the negro is mentally and physically inferior to the white, and, therefore, less capable to resist the invasion of disease, and to overcome its action when once developed. In addition, the negro has a hypo-cranial and thoracic development, and consequently a hypo-cerebral and pulmonary capacity when compared with the white.

Croupous pneumonia has been with us endemic, and that there has been some occult condition or conditions that have developed and maintained the essential cause of this disease, there can be no doubt. The action of this essential cause has been, at times, inoperative, at others active. Sometimes for months there would be no cases, when, all at once, without warning and without the least visible modification of the sanitation of the prisons or the hygiene of the convicts, five, ten, or fifteen cases would occur in a few weeks, sometimes four or five in one week. I have come to regard one case as a certain indication of many. These things happen, the pneumonia appearing and disappearing without the least visible reason. Season, however, seems to have some influence—May, June and August being the months in which they have usually occurred. There have been a series of cases also in September, once last year. October, November, December and January have been peculiarly exempt from these usual epidemics. There have been a great many cases in February, March and April—more in the latter than the two former—but the first months mentioned have been particularly fatal months. July ranks intermediate between the first three and the remaining months. Not only has June and August been the months of greater frequency, but also of greater fatality.

The cases occurring in the fall and winter months have been less fatal.

### CLINICAL HISTORY.

There have been many peculiarities in the clinical history of this disease in many of these cases, when compared with the text-books. Pain, cough and rusty sputa have been absent in many cases—sometimes one, sometimes all three. In many cases, particularly those in which pain was absent, the crepitant rale was not heard. In many cases the evolution of the inflammation was delayed for several days, from three to six, as determined by the physical signs.

The most characteristic symptom, and the one always present, was the extremely sick condition of the patient. All were characterized by great prostration, muscular debility and general weakness. As a rule, the onset of the disease has been sudden, without premonition. Generally a chill, more frequently chilly sensations, then a rigor, occurring usually at night, followed by high fever, fast breathing, general prostration and apparent gravity of disease. Pulse, 120 to 140; temperature, 104° to 106°; respiration, from 36 to 50. Constipation the rule. Nausea or vomiting rare. Delirium has been rare. Wakefulness and restlessness the rule. The absence of pain and cough I account for by the absence of the consecutive bronchitis and pleurisy. The rusty sputa, when absent, simply demonstrates that there is no hemorrhage in the exudate, and but few red corpuscles; or it may be, in those cases where there was no cough, the absence of the latter explained the want of the rusty sputa. Nearly all, however, had cough before the end of the case, and the majority pain also. But in many cases there was no rusty sputa, and in some sputa of no kind.

The physical signs in many cases were negative for several days. In some cases there were slight signs of disease, a slightly elevated pitch of the percussion note, a slightly diminished respiratory murmur, with a very slight broncho-vesicular quality, a slightly increased vocal resonance, with, as a rule, no increase of vocal fremitus, characterized the delayed cases. In others, the physical signs were pronounced and typical from the beginning. In those cases in which the physical signs denoted delayed evolution of the inflammatory process, the disease was longer in duration, and more fatal than the more typical cases.

In the majority of the cases that recovered, crisis was the termination—a few by lysis. In most instances, simultaneously with the end of the constitutional symptoms, or very soon after, resolution set up. In some the physical signs remained unchanged for two days, and in a few cases for weeks, and in two cases two or three months.

The portion of the lungs involved, and the extent of the lesion, differs widely in recent and former years. In the first fifty cases, only a small percentage were double, and, as a rule, a lower lobe, particularly the right, was the seat of the local lesion. After this, the tendency to double pneumonia and to involve the upper lobes increased, so that during the last four years over 50 per cent. of the cases were double, and nearly 75 per cent. involved an upper lobe, generally the right, and in a few cases the entire lungs were simultaneously involved, death closing the scene in twenty-four to thirty-six hours. In others the lobes of both lungs were successively attacked, so that at the post mortem all the stages of the disease could be demonstrated. In a few cases the patient, however, recovered —resolution successfully following the invasion.

Complications were also more frequent during recent years. Meningitis, pericarditis and cardiac thrombosis were the most frequent complications. In many, the disease occurred in those affected with chronic diseases—rheumatism, organic heart disease, scrofula, etc. I do not believe, however, that these diseases predisposed to the development of the disease, but, of course, increased the fatality.

MORTALITY.

I can give only accurate data up to October, 1889, as I have not had the time to compile the statistics during the last two years. Up to October, 1889, there were 155 cases and 50 deaths, or a mortality of 32.25 per cent. Since October, 1889, the relative mortality has been a little less, which would make the mortality of the two hundred cases about 30 per cent.

Studying this mortality for different years it ranges from 12 to 30 per cent. thus demonstrating the difference in the severity of the various series of cases.

In nearly all these cases severe complications were present and generally the pneumonia was double, and in the vast majority the upper lobe of the right lung was involved. In most of the cases the upper and lower lobe of the right lung and the lower lobe of the left. In only a few was the middle lobe of the right the seat of disease. Meningitis and pericarditis (not speaking accurately) was present, each in 4 or 5 cases.

In one case both were present. Cardiac thrombosis, or heart-clot, almost always present, the physical characters of which demonstrated in many instances that it had existed for a considerable time, and was not developed while the patient was in articulo mortis or post mortem. In nearly all there were old adhesions, in some, throughout, in most of them, posteriorly, demonstrating previous attacks of pleurisy or pneumonia. In the majority there were a few recent adhesions, and generally the pleura was covered with lymph corresponding to the inflamed pulmonary tissue, particularly that part of the lung in which the pneumonia was in the second or third stage, rarely when in the first stage. In some there was no signs of pleurisy whatever, either old or recent, and the lungs could be turned out like a liver.

The appearance of the lung itself depends of course upon the extent and stage of the inflammation. The majority died during the second stage of a whole lung or one lobe of each lung, with the first stage in one or more lobes, sometimes the whole lung.

Purulent infiltration rare; abscess nor gangrene has been observed. Œdema always present, usually confined to the pneumonic area, but not infrequently the healthy lung, when there was any, was also œdematous. In a few instances the pneumonic inflammation was in the third stage, resolution progressing, the patient dying from the complication. In two cases the patient entirely recovered, so far as the pneumonia was concerned, subsequently dying from pulmonary infarction and embolism of the arteria innominata. Heart clot existed in both of these cases. One portion of the clot in the right ventricle was dislodged and conveyed to the lungs and gave rise to a series of attacks of embolic pneumonia, with clinical history resembling intermittent fever, which diagnosis was first made, finally dying from exhaustion. The lungs were full of embolic consolidations and an old firm clot in the right ventricle. In the other, resolution was complete, the heart remaining fast and irregular, when the patient suddenly became violently ill with symptoms of dyspnœa and coma and died. Post mortem showed a firm clot easily removed from the arteria innominata and right subclavian and common carotid arteries. Resolution in both lungs complete.

I will not worry you with a further analysis of these cases, but will proceed to discuss some points which may be of interest.

The great prostration is undoubtedly largely of nervous origin and is due largely to the essential cause of the disease. The violence of the symptoms in those cases in which the physical signs are practically negative for several days, showing that there is no active exudative inflammation at that time, and the further fact that physical signs show solidified lung, often after all constitutional symptoms have subsided, demonstrate the constitutional nature of the disease and that the constitutional symptoms are not merely the *expression* of a local process. Yet according to my experience in the majority of cases, the constitutional, as well as local symptoms, are more severe in those cases with extensive local lesion—not always *pari passu,* but approximatively. I therefore cannot agree with those authors who teach that there is no connection or parallelism between the local inflammation and the general symptoms It is true that cases involving only one lobe are sometimes unusually severe, but I have never seen a case of double pneumonia in which the constitutional symptoms were mild. In those cases in which the pneumonia is central, that is, invades the pulmonary tissue from centre to periphery, I have found to be the most dangerous. These are the delayed cases and those in which we have the physical signs enumerated above.

My explanation of this is, that there is really more vesicular surface involved than when the disease commences on the periphery. The air cells are smaller than at the margins, borders and periphery of the lungs, thus in a given cubic area of lung, multiplying the diameters and consequently the circumferential area, and hence the aggregate vesicular surface becomes increased. Therefore, in a given cubic area of lung in which the cells are smaller, there is more vesicular space than in a like area, where the cells are larger.

This being true, the inflamed surface is much greater and the respiratory function correspondingly embarrassed. I found in these cases of central pneumonia in which the exudation was fully developed, that portions of tissue cut from the margins and periphery would not sink in water and showed signs of crepitation On the other hand, in those cases in which the periphery was first involved there were no signs of crepitation there, and portions cut from it would sink in water, and showed some signs of crepitation.

This explanation, of course, contradicts the idea that a whole lobe is alike involved, and to this latter idea I am inclined, because it is very rare that a whole lung or whole lobe, notwithstanding it is involved in the pneumonic process, even in the second stage, will sink in water, or that does not show circumscribed areas of slight crepitation.

I therefore believe, that in central pneumonia, for the reasons given, there is a greater extent of vesicular surface involved. It is in these central cases that the crepitant rale is often absent and in which there is often no consecutive pleurisy.

I am, therefore, inclined to the belief that the crepitant rale is often caused by the pleurisy, as some teach, and not by the separation of the adhered vesicular surfaces. The absence of pleurisy also explains in these delayed cases, the absence of pain, and possibly cough, also, when it is absent. In fact, I believe the cough is mainly due to the consecutive bronchitis, as well as most of the expectoration and the pain, as all agree, to the pleurisy.

Heart clot I think, is more than a mere sequence of articulo-mortis or post-mortem changes. I believe it is often a veritable complication and is in many cases the immediate cause of death. That is to say, that many cases would recover were it not for the formation of these clots. I know that some of the clots in my possession ante-dated death by several days. They were firmly attached to the columnæ carnæ and chordæ tendinæ and musculi pectinati, often extending into the aorta and pulmonary arteries ; in a few instances into even the innominate artery. They can be pulled apart like the layers of an onion, in fact, possess all the characteristics of thorough ante-mortem clots.

There can be no doubt in my mind as to their often being the direct cause of death, in otherwise favorable cases.

In corupous pneumonia we have all the essential conditions for the formation of heart clots, which I will not take up your time to enumerate.

The lungs of convicts who worked in mines were blacker than the normal or pathological condition in pneumonia. This is due to deposit of coal dust in the connective tissue, a kind of latent anthracosis. This condition, in my opinion, predisposes to an increase in the extent of the pulmonary lesion and also crippled the lung in such a way as to modify unfavorably its capacity to recover. Therefore, we can expect a larger mortality among a mining population who have pneumonia.

The prognosis of pneumonia depends upon the character of the epidemic for mildness or severity; the presence or absence of complications, the type of the disease and, in my opinion, very important, the extent of the pulmonary lesion.

The treatment of croupous pneumonia is up to this time a disappointment. There is no specific for the disease, no antiseptic that will destroy the essential cause when active in the body, no system nor plan nor method, consisting of various features, whether sedative, stimulant or mixed as yet devised to treat this disease with success.

I know of no method that will abort or even modify the pulmonary inflammation. These views are pessimistic, but unfortunately true. Notwithstanding, life can sometimes be saved by judicious treatment. In estimating the results of treatment, numerical statistics alone prove nothing. Unless the nature of the epidemic, the extent of the local lesion and the presence or absence of complications and their nature are fully understood and their influence estimated, mere statistics and percentages are worthless.

My experience has taught me that the mild uncomplicated cases and of limited extent, say one lobe, preferably a lower lobe—intrinsically tend to recovery, and nearly all will get well, barring the influence of age and bad physical condition, etc., with or without treatment. On the other hand the severe cases, characterized by high fever, fast pulse, fast and imperfect respiration, with considerable dyspnœa and great extent of tissue involved, say one lung or one lung and one lobe of the other, especially if complicated, intrinsically tend to death, and the majority will die with any treatment whatever. In my opinion, aconite, veratrum, purging, tartar emetic and any and all depressing agents have no place in the treatment of this disease. The main object in treatment should be to modify the depressing effects of the morbific influence, which is best done by the judicious administration of opium, to reduce the temperature, which is best done with large doses of quinine, from 20 to 40 grains given at once; to sustain the vital powers with alcoholic stimulants and plenty of nutritious diet. Medicinal stimulants may also be given, of which digitalis stands at the head of the list, strychnine next. According to my experience carbonate of ammonia is useless, but I generally give it as a matter of routine. The local treatment is very simple. Turpentine stupes, or any mild counter irritation will answer—enveloping the chest with cotton, over which oil silk is neatly fastened, will do in cases of children. Blisters, in my judgment, have no more influence in modifying the extent or course of the inflammation, than they would the lesions of Peyer's patches in typhoid fever. Blisters, therefore, in my opinion have no place in the treatment of this disease.

Special symptoms and conditions must, of course, be met. Œdema is often an immediate cause of death, and can sometimes be relieved. This is best done by extensive dry cupping and an increase of the stimulants. Bronchitis when present should be appropriately treated with stimulating expectorants.

I will not consume more of your time in discussing the treatment of this disease.

In concluding allow me to impress upon your mind that croupous pneumonia is a general, constitutional, infectious disease, with local lesions in the pulmonary parenchyma, and that the usual treatment for true inflammation is not applicable to the treatment of this disease.

